# Postoperative Intensity-Modulated Radiation Therapy for Myoepithelial Carcinoma in the Parotid Gland

**DOI:** 10.7759/cureus.21197

**Published:** 2022-01-13

**Authors:** Kanako Nakatsu, Takahiro Kishi, Junko Kusano, Yasuyuki Hiratsuka, Takashi Ishigaki

**Affiliations:** 1 Radiation Oncology and Image-Applied Therapy, Kyoto University Hospital, Kyoto, JPN; 2 Radiation Oncology, Osaka Red Cross Hospital, Osaka, JPN; 3 Otolaryngology/Head and Neck Surgery, Osaka Red Cross Hospital, Osaka, JPN

**Keywords:** nivolumab, postoperative radiation therapy, parotid gland, myoepithelial carcinoma, intensity-modulated radiation therapy

## Abstract

Myoepithelial carcinoma (MC) is an extremely rare form of tumor, with no standard treatment established to date. Although several reports have discussed postoperative radiation therapy (PORT), few have applied intensity-modulated radiation therapy (IMRT), and none has described the dose and radiation field in detail. In this report, we describe a case of MC of the parotid gland that was treated with high-dose IMRT (70 Gy) after partial resection. The patient, a 61-year-old female, underwent excisional surgery and was diagnosed with MC arising from a pleomorphic adenoma (PA). Postoperative irradiation was administered as 70 Gy in 35 fractions of local radiation. The patient had cancer recurrence in the irradiated field. However, no serious adverse events associated with the radiation therapy have been confirmed, implying that postoperative high-dose radiation therapy may be safely administered via IMRT.

## Introduction

Myoepithelial carcinoma (MC) is an extremely rare form of tumor that accounts for only approximately 1% of parotid carcinomas [1]. Radical surgery is often performed as the initial treatment for MC, after which adjuvant therapy is considered. However, due to the rarity of MC, no standard treatment has been established to date [1,2]. Radiation therapy (RT) is an important postsurgical adjuvant therapy; however, its efficacy for MC is debatable, and adverse events must be avoided. In this context, intensity-modulated RT (IMRT), which has been widely applied in recent years, may be an appropriate form of treatment [3,4]. Although some reports have discussed the application of postoperative RT (PORT) for MC [5-10], few have described IMRT, and none has described the dose and radiation field in detail.

In this report, we present a case in which high-dose IMRT (70 Gy) was administered to a patient following MC resection in the parotid gland. The usefulness of RT and the appropriate dose and radiation field are discussed in the context of recent literature.

## Case presentation

**Figure 1:**
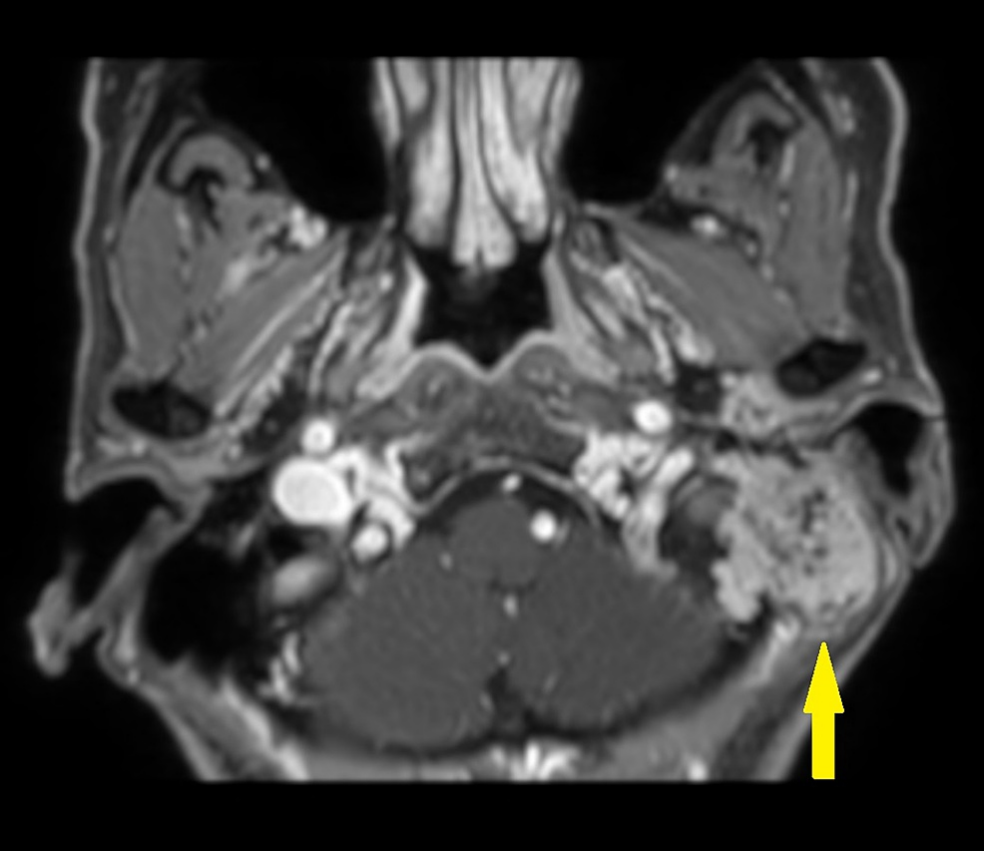
MRI at first visit Gadolinium-enhanced fat-saturated T1-weighted image Yellow arrow: tumor located in the left parotid gland

**Figure 2:**
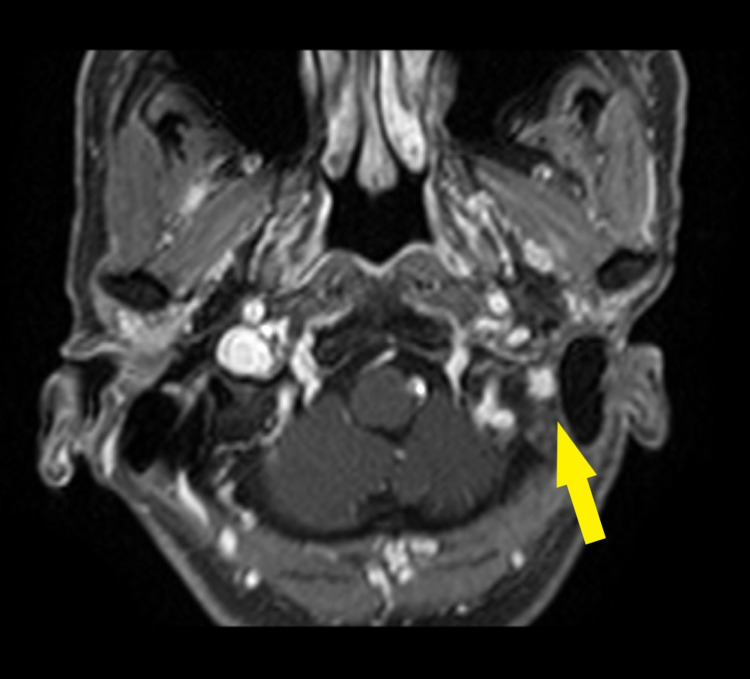
First recurrence in April 2018 Gadolinium-enhanced fat-saturated T1-weighted image Yellow arrow: local recurrence within the radiation field

**Figure 3:**
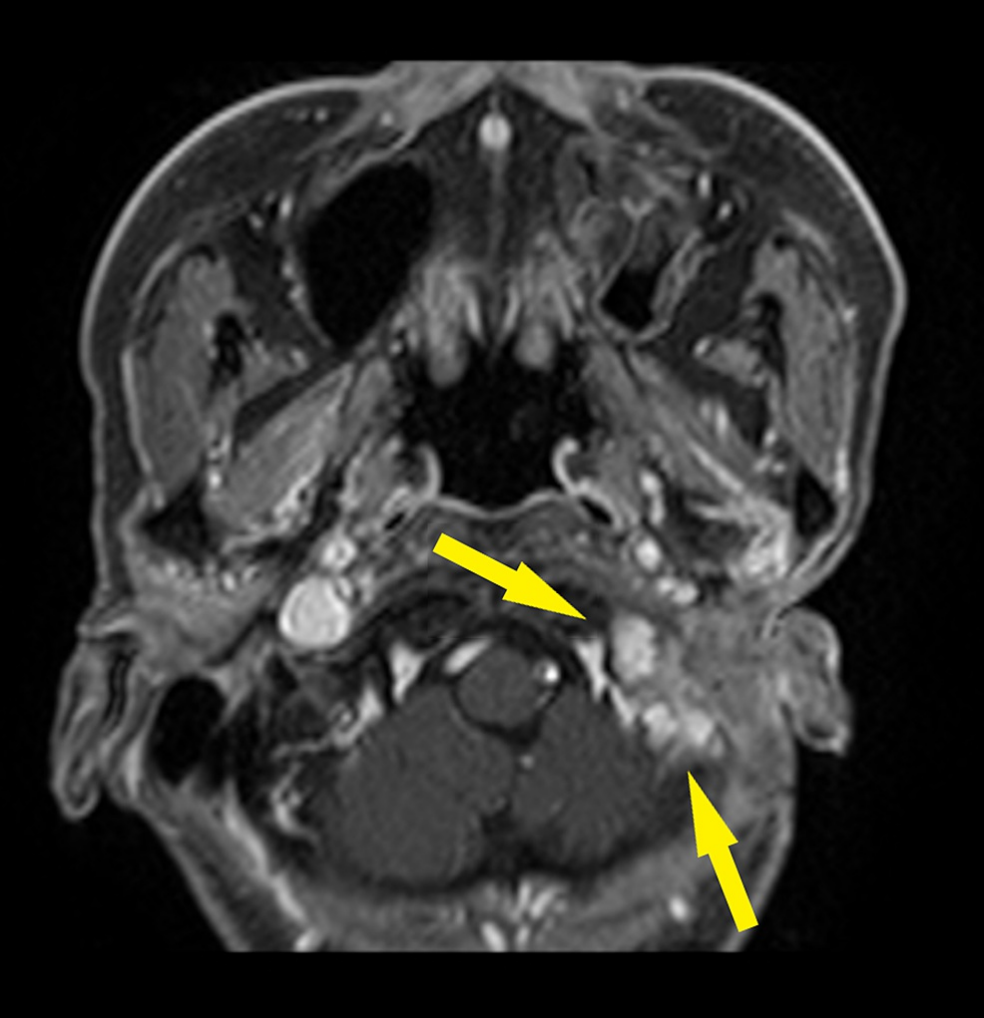
Second recurrence in March 2019 Gadolinium-enhanced fat-saturated T1-weighted image Yellow arrows: a lesion suggestive of recurrence

A 61-year-old female presented to our hospital in October 2016 complaining mainly of left ear obstruction and left facial numbness. She had undergone a partial left parotidectomy for pleomorphic adenoma (PA) in 2009 at another hospital. Imaging studies revealed a tumor located in the left parotid gland (Figure 1). The tumor had invaded the temporal bone and mandible and was suspected to have invaded the sigmoid sinus. Fine needle aspiration cytology was performed, and a diagnosis of PA was made. However, because the possibility of malignancy could not be ruled out, total resection of the left parotid gland and combined resection of the temporal bone and mandible were performed in January 2017. The histological diagnosis was myoepithelial carcinoma ex PA. The patient was diagnosed with positive resection margins on the cranial side of the posterior parotid gland and underwent localized postoperative IMRT (70 Gy in 35 fractions) from February to April 2017. Magnetic resonance imaging (MRI) in April 2018 showed local recurrence within the radiation field (Figure 2), to which 95%-100% of the prescribed dose was delivered. The patient underwent reoperation in August 2018 to remove the tumor. The facial nerve was preserved, and the tumor was excised without the creation of a gross residual lesion. However, as the tumor was divided into small fragments, the margins were difficult to assess. In March 2019, MRI again showed a lesion suggestive of recurrence (Figure 3). No biopsy was performed, but recurrence was clinically diagnosed based on the disease course. TC therapy consisting of six cycles of three weekly doses of carboplatin (570 mg/body weight; AUC = 6) and paclitaxel (175 mg/m^2^) was started in March 2020. MRI in June 2020 showed progressive disease, and nivolumab (twice weekly, 240 mg/body) was started in July 2020. At present, the tumor is growing slowly, although the rate of growth has slightly decreased.

Histopathological features

Histopathological analysis showed PA with myxoid stroma, intermixed with myoepitheliomatous and squamous epithelial-like components, and concentric keratinization. Squamous cell or adenoid cystic carcinoma components were not observed in the specimen. Cellular atypia was inconspicuous, and the Ki-67 labeling index was less than a few percent. Although these findings were atypical for MC [11], the cancer was diagnosed as carcinoma ex PA with mainly epitheliomatous components due to the infiltrative growth. Immunohistochemical analysis of the tumor showed positive reactivity to AE1/AE3, CK7, EMA, SMA, S-100, GFAP, and p63 and negative reactivity to CK20.

IMRT planning summary

**Figure 4:**
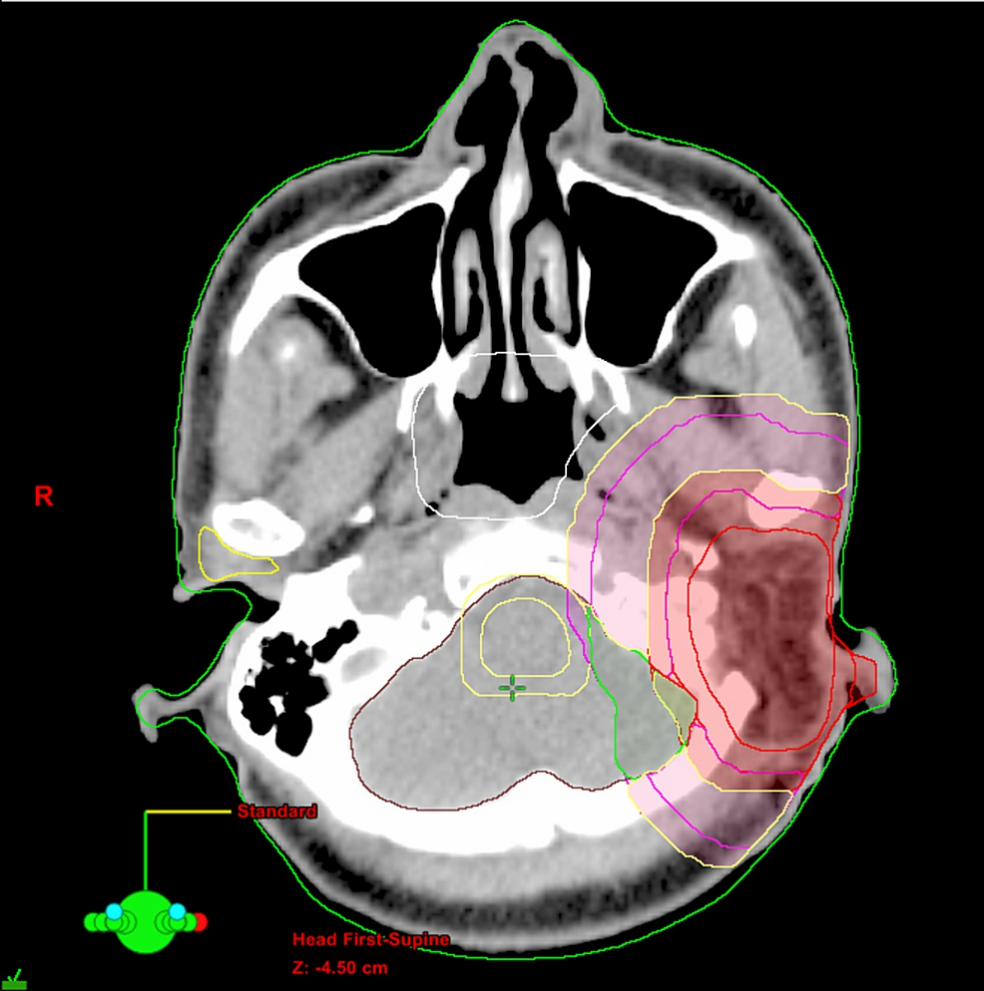
Target and avoidance structures Example axial slice representing the method used for contouring target and avoidance structures Red: old GTV; inner magenta: CTV1; inner yellow: PTV1 (mod); outer magenta: CTV2; outer yellow: PTV2

**Figure 5:**
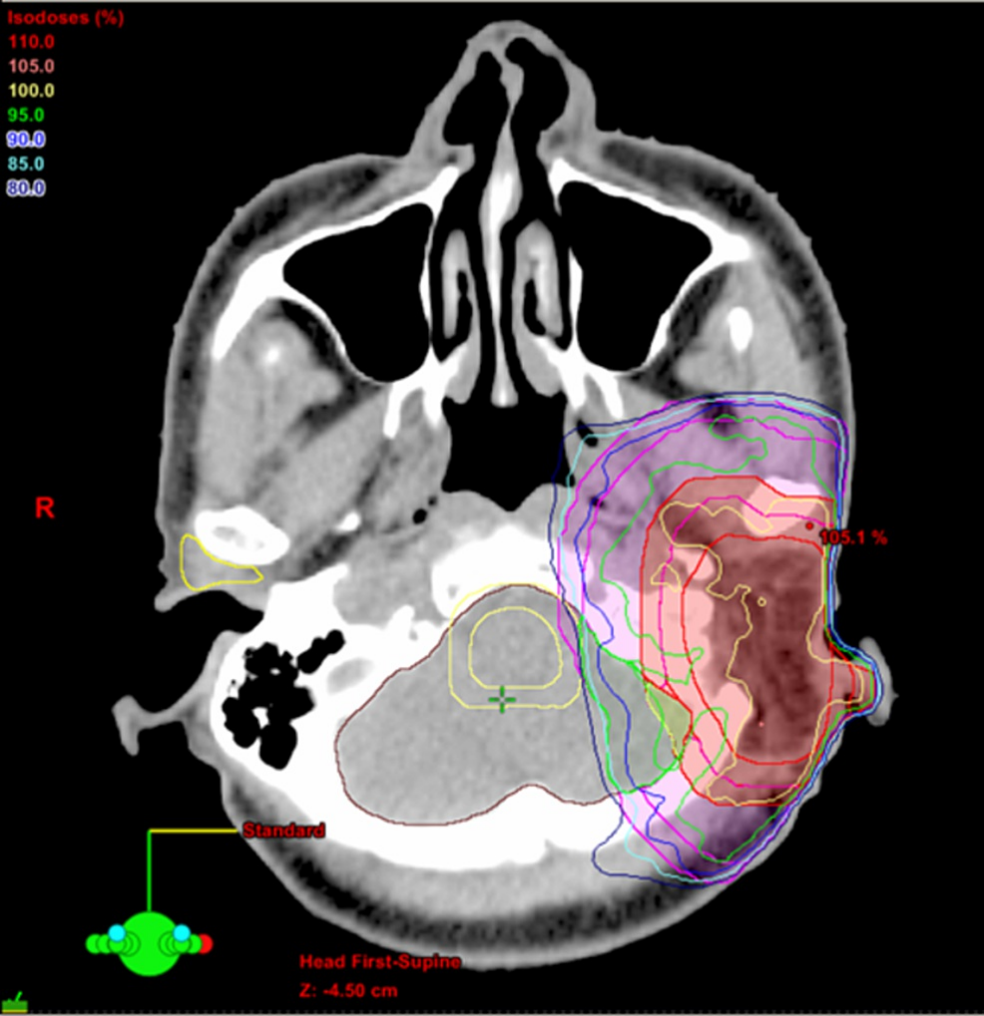
Representative axial sections with dose distributions

**Figure 6:**
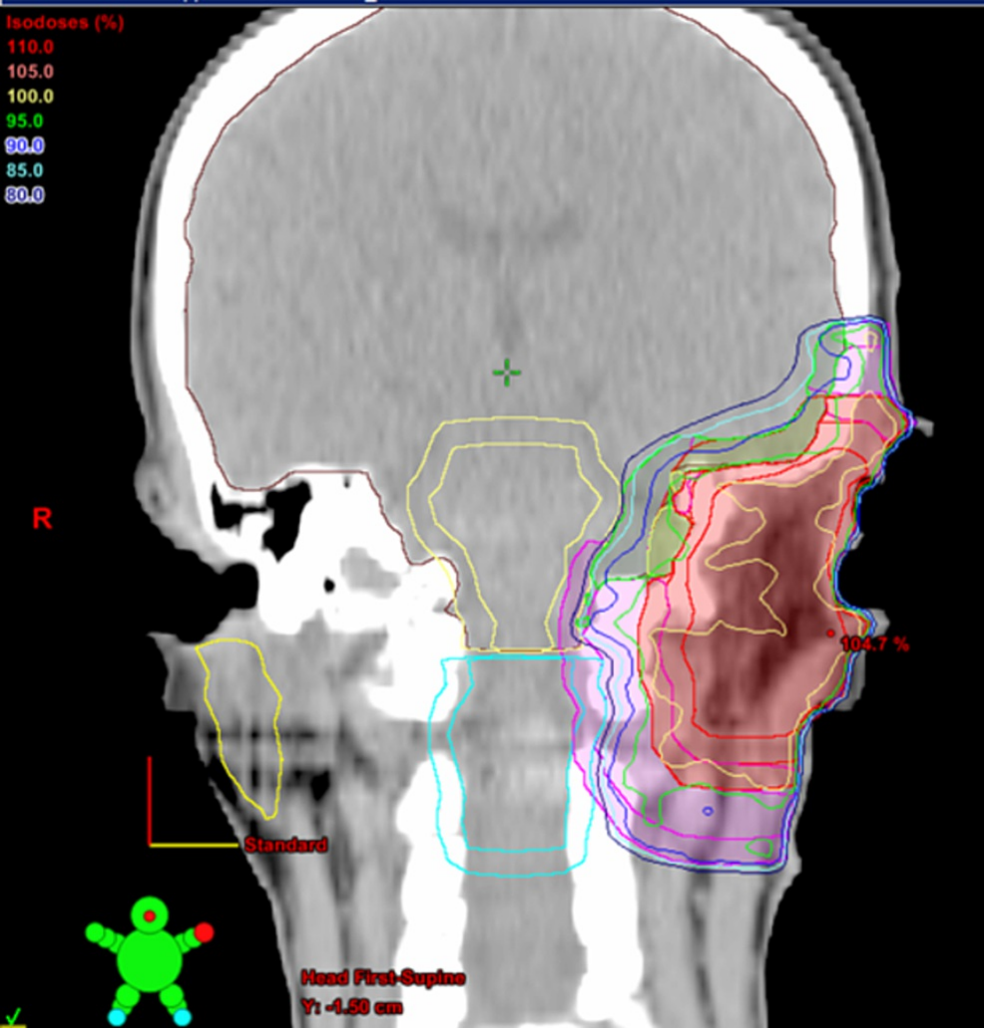
Representative coronal sections with dose distributions

**Figure 7:**
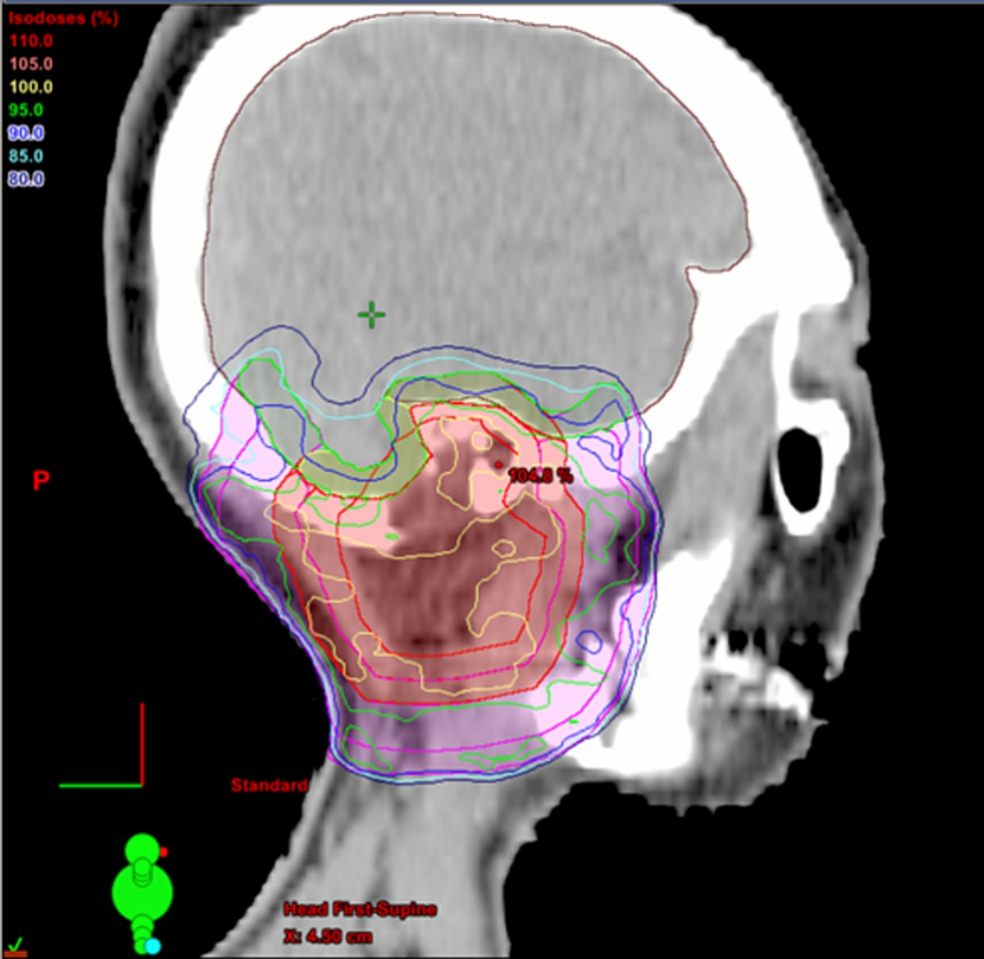
Representative sagittal sections with dose distributions

The patient underwent computed tomography (CT) scanning (slice thickness: 2.5 mm) in the supine arm-down position with a thermoplastic immobilization mask around the head and neck. Contours were developed using preoperative imaging (CT, MRI, and fluorodeoxyglucose positron emission tomography/CT) findings and the in situ contralateral parotid as a guide to contour a dummy structure representing the removed parotid gland and tumor (old GTV). This structure was expanded by 0.5 and 2 cm into the surrounding soft tissue to attain the clinical target volumes (CTV1 and CTV2, respectively). The expansion was reduced at the natural barriers to tumor extension (e.g., where the skull and intracranial structures overlapped with the region 2 cm distant from the old GTV, the skull was included in the CTV1, but the intracranial structures were not). The planning target volumes (PTV1 and PTV2) were created by uniformly expanding both CTV1 and CTV2 by 5 mm. We defined PTV1mod as a structure excluding the part of the brain that overlapped PTV1. The prescription doses to PTV1mod and PTV2 were 2.0 and 1.8 Gy per fraction, for total doses of 70 and 63 Gy in 35 fractions with a simultaneous integrated boost, respectively. The dual arc volumetric modulated arc therapy (VMAT) plan was optimized using the Eclipse planning system (Varian Medical Systems, Palo Alto, CA, USA). The plan featured a photon energy of 6 MV without any bolus. The planned treatment included 280° arcs of VMAT (avoiding the gantry angles of 0°-40° and 320°-360°). The treatment goals were to deliver prescription doses to 50% of PTV1mod and ≥50% of PTV2. The area where PTV1 and the brain overlapped was optimized to receive as high a dose as possible without exceeding 70 Gy. The maximal hotspot was 106.8%, which was within the PTV. The doses to the organs at risk (OARs) are shown in Table 1, and the representative dose distributions are shown in Figure 4-7. The left cochlea received a high dose of radiation, but this was unavoidable because we prioritized target coverage. Image-guided radiotherapy was performed. We scheduled cone-beam computed tomography daily for the first three days and then once weekly and on-board imaging four times a week.

**Table 1:** Doses to organs at risk

Organ	Maximum dose (cGy)	Mean dose (cGy)
Brain	6834.6	1312.6
Brain stem	4829.6	2750.4
Spinal cord	4567.2	3242.3
Right cochlea	2156.3	1713.3
Left cochlea	7113.1	6795.6
Right parotid gland	2856.5	2192.1
Right submandibular gland	2197.9	1158.7
Oral cavity	4184.8	2458.5

Adverse events

Adverse events were evaluated according to the Common Terminology Criteria for Adverse Events v5.0. Acute adverse events included grade 1 (G1) dermatitis, G1 alopecia, G1 mucositis, G1 taste disorder, and G1 dry mouth. As late adverse events, G1 permanent hair loss was observed in the area irradiated with high doses. No other severe (≥G2) adverse events (e.g., facial nerve paralysis and auditory impairment) were observed.

## Discussion

MC, also known as malignant myoepithelioma, is an extremely rare form of tumor that accounts for only approximately 1% of parotid carcinomas [1]. Due to its rarity, no standard treatment has been established. However, case reports indicate that surgery is often performed as the initial treatment. No systematic review of the effectiveness of neoadjuvant chemotherapy has yet appeared. Since local recurrence is the most common form of recurrence, radical surgery is typically recommended. However, depending on the size and location of the tumor, it may be difficult to resect completely; a high recurrence rate has been reported for these cases [4]. Therefore, when complete surgery is not possible, some form of postoperative therapy may improve the outcome. However, there is no consensus on which method of postoperative therapy should be selected.

Xu et al. reported no significant difference in overall survival between RT and non-RT groups of patients with MC [2]. However, the breakdown of patients was not specified; therefore, there may have been more cases of incomplete surgery in the RT group. In fact, Giridhar et al. reported a significant decrease in the local recurrence rate and prolongation of progression-free survival in an RT group following R1 resection [1]. There have also been a few case reports of RT for MC (Table 2) [5-10,12,13].

**Table 2:** Case reports of radiation therapy for myoepithelial carcinoma AWD: alive with disease; BM: buccal mucosa; DOD: dead of disease; FM: floor of the mouth; IMRT: intensity-modulated radiation therapy; meta: metastasis; none: no recurrence nor metastasis; PORT: postoperative radiation therapy; R1: microscopic remains; R2: macroscopic remains; rec: recurrence

Reference	Primary site	Purpose	Dose/fraction	Method	Rec or meta	Observation period, outcome
[5]	Parotid	PORT	60 Gy, 30 fractions	Unknown	Meta (11 months after PORT)	16 months, AWD
[6]	Infratemporal fossa	PORT	60 Gy	Unknown	Rec	6 months, AWD
[7]	Sellar region	PORT (R2)	54 Gy, 30 fractions	3D conventional	Rec and meta (eight months after PORT)	20 months, DOD
[8]	Hard palate	PORT (R1)	Unknown	Unknown	Meta	3 months, AWD
[9]	Parotid	PORT (R2 resection)	Unknown	Unknown	Rec	9 months, AWD
[10]	Parotid 6, palate 3, BM 1, FM 1, nasal 1	PORT	Unknown	Unknown	Rec 5, meta 2, none 5	24 months (median), unknown
[12]	Soft palate	Definitive	62.5 Gy, 25 fractions	IMRT	None (tumor remains)	18 months, AWD
[13]	Submandibular	Definitive	70 Gy	Unknown	None	3 months, AWD

In summary, some reports have described tumor shrinkage following RT; however, there are few reports supporting the usefulness of postoperative RT. The factors predicting the success of RT are also unknown. Because the usefulness of PORT remains controversial, it seems necessary to avoid adverse events to the greatest extent possible when performing PORT. The risk of adverse events due to RT generally increases as the radiation dose increases; IMRT is a strategy used to reduce adverse events at the high RT doses required for head and neck cancer [3,4]. In cases of parotid adenocarcinoma, dose reduction to OARs has been achieved using IMRT [14]. Fang et al. discussed potential radioresistance and the short doubling time of MC and recommended an accelerated high-dose approach [15]. It is quite possible that IMRT will allow an increase in the prescribed dose to the tumor while reducing the dose to OARs.

In the present case, no significant late adverse events have been observed within the 3.5 years since RT completion, implying that IMRT allows safe delivery of high-dose radiation, even after surgery. It is also important to perform salvage surgery safely in some cases of recurrence after PORT, and IMRT may facilitate this. However, to date, there are no detailed reports of PORT using IMRT for MC in the literature.

MC is reported to have a low rate of lymph node metastasis [2], and prophylactic dissection as a routine procedure is rarely performed at the time of surgery unless there are prominent lymph node metastases [16]. Therefore, it may not be necessary to include the regional lymph node area in the radiation field. In the present case, only local irradiation was performed, and no lymph node metastasis has occurred to date. The relationship between the extent of RT and lymph node recurrence has not been reported in detail; therefore, we hope that the present report will contribute to determining the extent of RT in future treatments.

Finally, nivolumab was used in this case. Niwa et al. reported the results of a multicenter retrospective study [17]. Although the efficacy of nivolumab in patients with salivary gland carcinomas was limited, some patients achieved long-term disease control. Takemoto et al. and Nakamura et al. reported parotid cancers that responded to nivolumab [18,19]. However, none of these reports included MC cases. To the best of our knowledge, this is the first report on the use of nivolumab to treat MC. In our present case, although nivolumab did not reduce tumor size, it slowed tumor growth. It is thus possible that nivolumab contributed to disease control. Nivolumab is expensive, and its use is thus contingent on a reasonable expectation of efficacy. Further case studies, including verification of cost-effectiveness, are required.

## Conclusions

We report a case of postoperative high-dose irradiation (70 Gy) for the treatment of MC arising from the parotid gland. To date, the patient has not reported any serious late adverse events.

Although it may theoretically be desirable to administer high doses of radiotherapy for MC treatment, the benefits of radiotherapy are uncertain. Therefore, serious adverse events of radiotherapy should be avoided. This case report demonstrates the potential of high-dose IMRT in improving the safety of PORT.
